# O16 - Children with asthma and rhinitis are at a higher risk of asthma exacerbations than children with asthma only: longitudinal analysis

**DOI:** 10.1186/2045-7022-4-S1-O16

**Published:** 2014-03-14

**Authors:** Matea Deliu, Danielle Belgrave, Aida Semic-Jusufagic, Lesley Lowe, Angela Simpson, Adnan Custovic

**Affiliations:** 1 University of Manchester, Manchester, United Kingdom

## Background

There is a paucity of longitudinal data on the effect of rhinitis on asthma exacerbations during childhood. Within the context of unselected birth cohort (Manchester Asthma and Allergy Study), we investigated the association between rhinitis and asthma exacerbations from early childhood to school age.

## Methods

Subjects were recruited prenatally and followed prospectively, attending follow-up at ages 3, 5, 8, and 11 years. A validated questionnaire was interviewer-administered to collect information on parentally-reported symptoms (n=1051). At each follow-up, current rhinitis was defined as sneezing, runny or blocked nose in the absence of cold or flu within the last 12 months, and asthma as a positive response to at least two of the following: 1) current wheeze; 2) physician-diagnosed asthma; 3) use of asthma medication. We measured specific airway resistance (sR_aw_) using plethysmography (ages 5-11 years) and FEV_1_ by spirometry (5, 8 and 11). Information on prescribed medication and severe asthma exacerbations (ATS definition) was extracted from participants’ medical records. The effect of rhinitis on asthma exacerbations and lung function was investigated using longitudinal analyses.

## Results

A total of 356 children had asthma on at least one time point, of whom 198 had rhinitis. In the multivariate models adjusted for the use of intranasal and inhaled corticosteroids as well as antihistamines, asthmatic children with current rhinitis remained markedly and significantly more likely to have frequent asthma exacerbations (>2 per year; OR[95%CI], 12.0 [2.12-67.92], p=0.005, aOR, 4.75 [1.54-14.65], p=0.007). However, there were no differences in the longitudinal measures of FEV_1_ or sR_aw_ between asthmatic children with and without rhinitis, before or after adjustment for the use of rhinitis or asthma medication.

## Conclusion

In the longitudinal analysis throughout childhood, children with asthma and rhinitis were also almost five times more likely to experience severe asthma exacerbations compared to children with asthma only.

